# First case of two supernumerary markers derived from chromosome 5 and chromosome 8

**DOI:** 10.1186/s13039-022-00601-5

**Published:** 2022-06-27

**Authors:** Roberta Giansante, Chiara Palka Bayard De Volo, Melissa Alfonsi, Elisena Morizio, Paolo Guanciali Franchi

**Affiliations:** 1 Department of Medical Genetics, “G. D’Annunzio” University, Via dei Vestini 31, 66100 Chieti, Italy; 2 Department of Medical Genetics, “SS. Annunziata Hospital”, Chieti, Italy

**Keywords:** Supernumerary marker chromosome, Chromosome 5, Chromosome 8, In situ hybridization, Dysmorphic facial features

## Abstract

**Background:**

Small supernumerary marker chromosomes (sSMC) are additional centric chromosome fragments too small to be identified by banding cytogenetics alone. A sSMC can originate from any chromosome and it is estimated that 70% of sSMC are de novo, while 30% are inherited. Cases of sSMC derived from chromosome 5 (sSMC5) are rare, accounting for1.4% of all reported sSMC cases. In these patients, the most common reported features are macrocephaly, dysmorphic facial features, heart defects, growth retardation, hypotonia, and intellectual disability. Also sSMC derived from chromosome 8 are very rare and the phenotype of patients with sSMC8 is very variable. Common clinical features of the patients include developmental delay, mental retardation, intellectual disability, hypotonia, hypospadias, attention deficit hyperactivity disorders (ADHD), skeletal anomalies, dysmorphic facial features, and renal dysplasia. To the best of our knowledge, in literature there are no cases with coexistence of sSMC5 and sSMC8, so we reviewed the literature to compare cases with SMC5 and those with SMC8 separately. This study is aimed to highlight the unique findings of a patient with the coexistence of sSMC5 and sSMC8.

**Case presentation:**

We describe a female patient with two supernumerary markers derived from chromosome 5 (SMC5) and chromosome 8 (SMC8). The patient was born prematurely at 30 weeks with respiratory distress and bronchodysplasia. On physical examination she presented dysmorphic features, respiratory issues, congenital heart defect, developmental delay, and intellectual disability. The G-banded chromosome analysis on cultured lymphocytes revealed in all the analyzed cells a female karyotype with the presence of two supernumerary chromosomal markers and the array-CGH highlighted the region and the size of these two duplications. We also used the fluorescent in situ hybridization analysis (FISH) using painting of chromosomes 5 and 8 to confirm the origin of the two sSMC. So, the karyotype of the patient was: 48, XX, +mar1, +mar2.

**Conclusions:**

This is the first case with two markers: one from chromosome 5 and one from chromosome 8. Based on the data reported, we can affirm that the phenotype of our patient is probably caused mainly by the presence of the sSMC.

## Background

Small supernumerary marker chromosomes (sSMC) are defined as structurally abnormal chromosomes that cannot be identified or characterized unambiguously by conventional banding cytogenetics alone and are equal in size or smaller than a chromosome 20 of the same metaphase.

sSMC are reported in 0.043% of newborn infants, 0.077% of prenatal cases, 0.433% of patients with intellective disability and 0.171% of subfertile people. About 70% of the cases are de novo, while 30% segregate within a family.

sSMC are a morphologically heterogeneous group of structurally abnormal chromosomes: different type of inverted duplicated chromosomes (inv dup), minute chromosomes (min) or ring chromosomes (r) [1].

In general, little is known about the exact mechanism of sSMC formation. Mainly, when, why, and how during gametogenesis or embryogenesis an sSMC evolves is unclear. Nonetheless, for all kinds of sSMC shapes there are models for how they could be formed. These ideas are based in part on the finding that uniparental disomy and sSMC can show up together and on the observation that sSMC can evolve by incomplete trisomic rescue. Overall, sSMC is formed by the combination of one or more rare events happening during gametogenesis or embryogenesis [2].

Apart from the correlation of about one third of the sSMC cases with specific clinical picture i.e., the *isochromosome-18p* (= i(18p)), *derivative chromosome 22* (der (22)t(11;22) (q23; q11.2) and the *cat-eye syndrome* (inv dup (22)), most of the remaining sSMC have not yet been correlated with clinical syndromes. Of this group, half of sSMC have derived from chromosome 15, although 50% of the carriers of sSMC(15) are healthy. However, only 8% of the carriers of sSMC derived from all other chromosomes, showed no clinical symptoms [3].

Cases of sSMC derived from chromosome 5 (SMC5) are rare and make up 1.4% of all reported and characterized SMC cases. In these patients, the most common features reported are macrocephaly, dysmorphic facial features, heart defects, growth retardation, hypotonia, and intellectual disability [4].

Cases of sSMC derived from chromosome 8 are rare. The phenotype of patients with sSMC(8) ranges from almost normal to variable degrees of abnormalities. Common clinical features of the patient included developmental delay, mental retardation, intellectual disability, hypotonia, hypospadias, attention deficit hyperactivity disorder (ADHD), skeletal anomalies, dysmorphic facial features, and renal dysplasia [5].

## Case presentation

We present the case of a girl born prematurely at 30 weeks with hospitalization in Neonatal Intensive Care Unit due to respiratory distress and bronchodysplasia. We have little anamnestic data at the time of birth because today the child is entrusted to an educational community. The presence of a small FOP and an iron-deficiency microcytic anemia under treatment was immediately found. At the age of 21 months on physical examination the patient had a height of 80 cm (at the 15th percentile) and a weight of 11.5 kg (between the 50 and 75th percentile). The dysmorphic features include macrocephaly, up-slanted palpebral fissures, hypertelorism, depressed nasal bridge, midfacial hypoplasia and heart-shaped mouth.

She also presented obesity and disproportion between the trunk and limbs length.

The G-banded chromosome analysis (450–500 band level) on cultured lymphocytes of the proband revealed in all the analyzed cells a female karyotype with the presence of two supernumerary chromosomal markers.

**Fig. 1:**
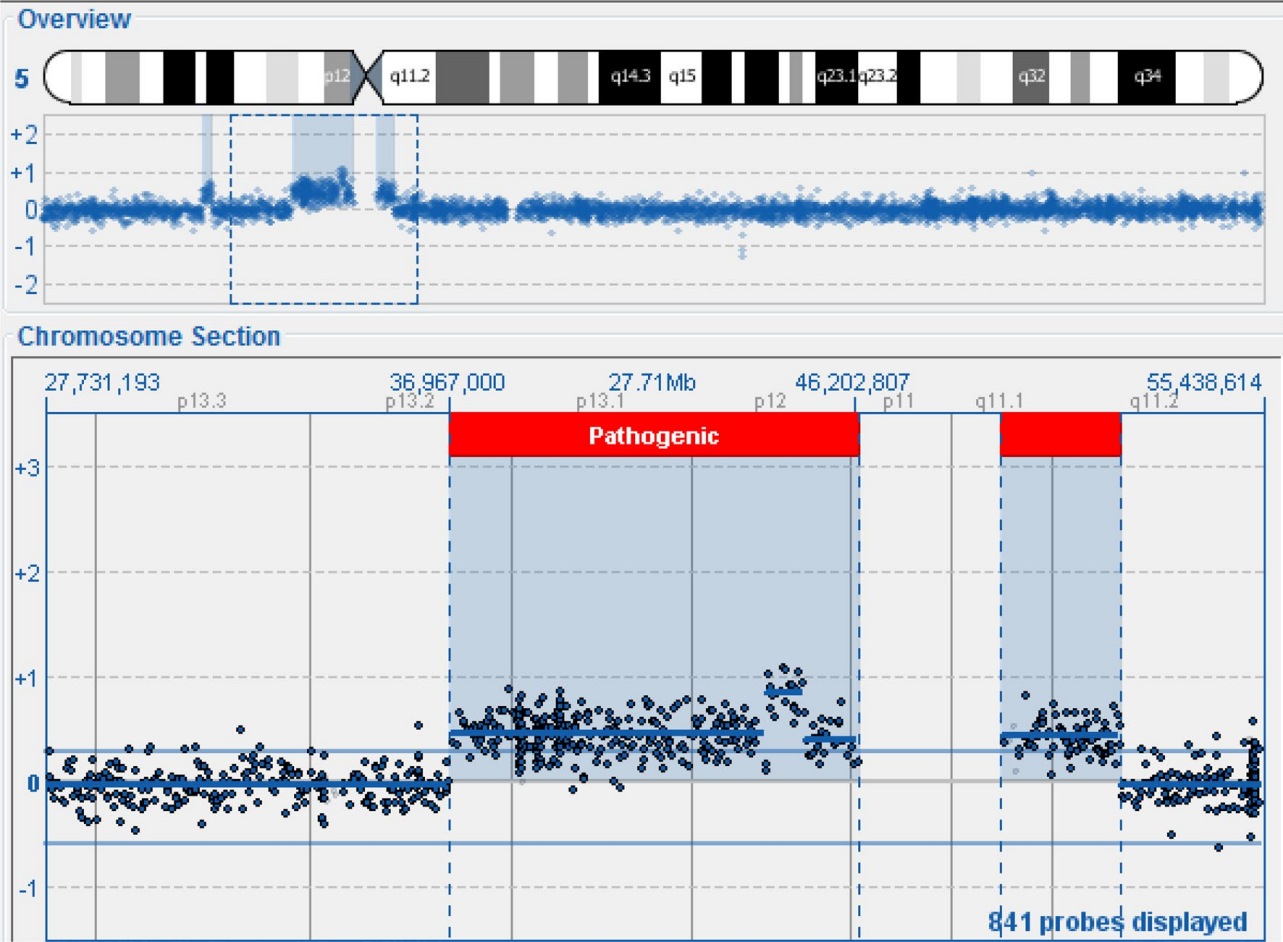
Array-CGH chromosome 5

**Fig. 2:**
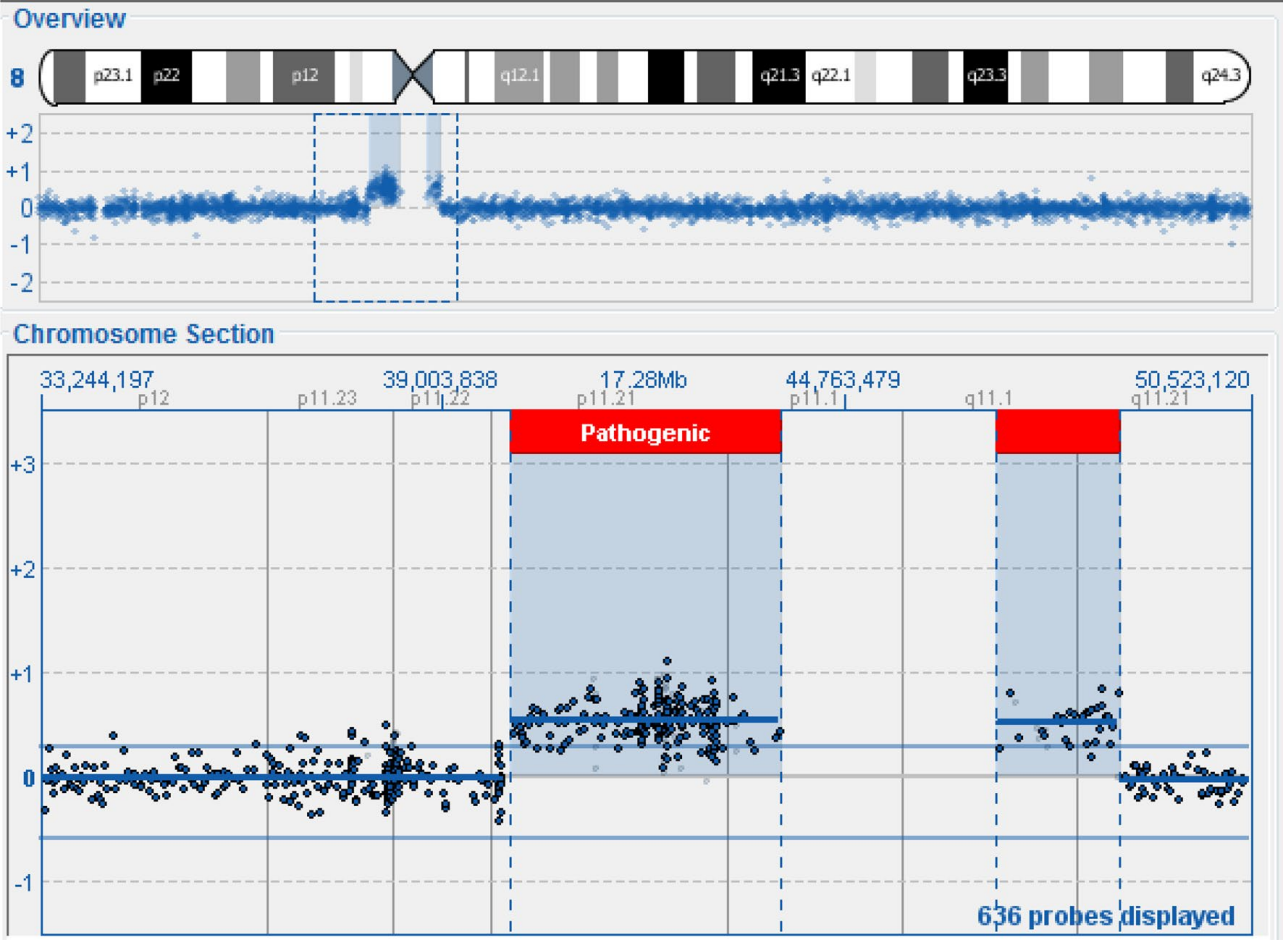
Array-CGH chromosome 8

The array-CGH was carried out using the 300 Kb resolution 4 × 180 k CytoSure Oligo OGT (Oxford Gene Technology) according to the recommendations of the manufacturer. The analysis highlighted the region and the size of these duplications:

1. 1.53 Mb duplication on chromosome 5p14.2p14.1, of uncertain significance,
2. Small supernumerary marker chromosome (sSMC) **5p13.2q11.2-5q11.1q11.2** which determines the presence of an 11.87 Mb duplication of this chromosomal region, of pathological significance (Fig. 1),
3. Small supernumerary chromosome (sSMC) **8p11.21p11.1-8q11.1q11.21** which determines the duplication of about 5.55 Mb of this chromosomal region, also of pathological significance (Fig. 2).

**Fig. 3:**
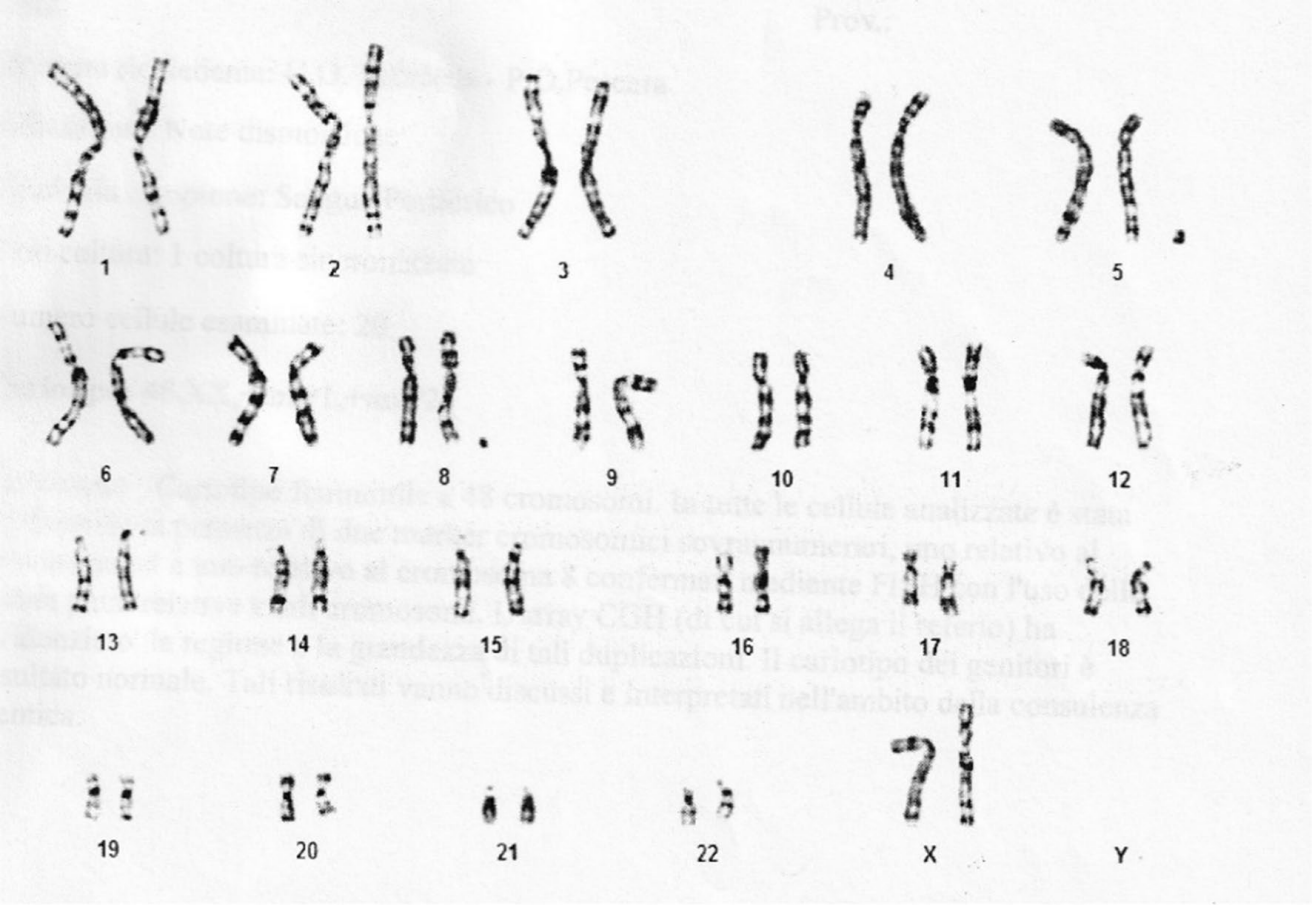
Karyotype 48, XX, +mar1 dn, +mar2 dn.ish r(5)(p13.2q11.2)(wcp5+), r(8)(8p11.21q11.21)(wcp8+).arr[GRCh37] 5p14.2p14.1(23,788,614–25,320,290)×3,5p13.2p11-5q11.1q11.2(36,967,000–52,194,364)×3,8p11.21p11.1-8q11.1q11.21(39,963,778–48,649,507)×3

The fluorescent in situ hybridization analysis (FISH), using painting of chromosomes 5 and 8 *(Kreatech, Resnova, Rome, Italy),* according to the manufacturer’s instructions, confirmed the origin of the two sSMC to be from chromosome5 and chromosome 8. So, the karyotype of the patient was as the following: 48, XX,+mar1 dn,+mar2 dn.ish r(5)(p13.2q11.2)(wcp5+), r(8)(p11.21q11.21)(wcp8+).arr[GRCh37] 5p14.2p14.1(23,788,614–25,320,290)×3,5p13.2p11-5q11.1q11.2(36,967,000–52,194,364)×3, 8p11.21p11.1-8q11.1q11.21(39,963,778–48,649,507)×3 (Fig. 3).

Parental karyotypes were normal.

The patient underwent further diagnostic tests:

Thyroid function, vitD and parathyroid hormone levels and screening for celiac disease were also evaluated, all of which were normal.

The abdominal ultrasound did not reveal any abnormalities in the abdominal organs.

The electroencephalography (EEG) in the predominantly agitated waking phase recorded a background activity of 5–6 c/s, structured for age, symmetrical on both hemispheres. No graph elements of certain pathological significance were recorded.

Brain magnetic resonance imaging (MRI) revealed a signal accentuation in the white matter adjacent to the left ventricular trigon, slightly wider than the contralateral, in relation to non-specific leukopathy.

An echocardiogram showed the absence of the FOP present at birth and overall, the echocardiographic findings are normal.

The neuropsychiatric evaluation revealed that the average age of development is about 15 months versus 25 months of chronological age (QS = 60).

In conclusion, the patient has a global developmental delay with an important speech delay.

Patient has recurrent respiratory infections (IRRs), and she performed the sweat chloride testing for diagnosis of cystic fibrosis (CF) which resulted negative. The otolaryngology visit highlighted the presence of adenotonsillar gigantism with severe Apnoic Respiratory Obstructive Syndrome and bilateral endothympanic effusion with flat tympanogram (type B).

The patient underwent antibiotic and cortisone therapy which improved her respiratory condition.

## Discussion

The peculiarity of our case is the coexistence of two supernumerary chromosomal markers derived from chromosome 5 and 8.

We use the online database Liehr T. [6] “Small supernumerary marker chromosomes” [6] where we found all the sSMCs present in literature, divided by chromosome. Thank to this database, we can find out two cases with multiple supernumerary markers, one case with 3 sSMCs and one case with 4 sSMCs.

The first case is characterized by the presence of three supernumerary markers derived from chromosome 5, 8 and 9. The patient is a 4-year-old male, and the clinical presentation is characterized by: mental retardation, hypertelorism, up-slanting palpebral fissures, large ears, bifid uvula, hypospadias, right testicular ectopia, syndactyly of 2–3 toes and obesity. His karyotype is the following: 49,XY,+3mar[9]/48,XY,+2mar[25]/47,XY,+mar1[13]/47,XY,+mar2[14]/46,XY[3].

The second case is characterized by the presence of four supernumerary markers derived from chromosomes 4,5, 8 e 13. The patient is a 7-years-old female, with mental retardation, developmental delay, facial abnormalities, abnormal placed anus and ASD. Her karyotype is the following: 47,XY,+mar1[4] 48,XY,+mar1,+mar2[13]/49,XY,+mar1,+mar2,+mar3[27]/50,XY,+mar1,+mar2,+mar3,+mar4[4].

Besides these two cases, there are no other cases with supernumerary markers derived from chromosome 5 and chromosome 8, so the markers of our case are examined individually and compared with some of the reported cases in literature.

We reviewed the literature for additional cases of SMCs involving overlapping chromosomes 5 material. The individuals in these cases possess features that vary widely (Table 1).

**Table 1:** Genetic and clinical features of cases with sSMC 5

	Avansino et al. [7]	Stankiewicz et al. (case 1) [8]	D’Amato Sizonenko et al. (case 1) [9]	D’Amato Sizonenko et al. (case 2) [9]	Sarri et al. [10]	Loscalzo et al. [11]	Melo et al. [12]	Hadzsiev et al. [13]	Camerota et al. [14]	Armstrong et al. [4]	Present case	Total
SMC5 coordinates	5p10 p13.1	5p14 q11.2	5p10 p13.3	5p13.3q12.3	5p13q11.2	5p11 p13.3	5p11q12.1	5p14 q11.1	5p13 q12.2	5p13.3 q11.2	5p13 q11.2	
Karyotype	47 XY+mar	47,XX,+r/46,XX	47,XX+r/46,XX	47,XY+r/46,XY	47 XX,+r/46,XX	46,XX,dup(5)	47 XX,+r/46,XX	47 XX,+mar	47 XX+r/46 XX	47 XY,+mar	48, XX,+mar1,+mar	
Age at evaluation	5 months	7 y	3 y	9 months	9 y	5 y	4 y	10 y	17 y	18 y	1 months	
Gestational age	31 weeks	NR	37 weeks	37 weeks	37 weeks	33 weeks	NR	At term	At term	30 weeks	30 weeks	
Developmental delay	+	NR	+	+	−	+	+	+	−	+	+	8/11
Intellectual disability	NR	+	+	−	+	NR	+	+	−	+	+	7/11
Congenital heart defect	+	−	−	−	+	+	−	−	−	−	+	4/11
Respiratory issues	+	−	+	+	−	+	−	−	−	+	+	6/11
Macrocephaly	+	NR	+	+	+	+	NR	+	+	+	+	9/11
Upslanted palpebral fissures	+	+	+	+	−	+	NR	−	+	−	+	7/11
Hypertelorism	+	+	+	+	+	+	+	+	+	−	+	10/11
Midface hypoplasia	+	−	+	+	−	−	NR	+	−	+	+	6/11
Depressed nasal bridge	+	+	+	+	−	+	−	+	+	−	+	8/11

We excluded the case reported by T. Liehr et al. [6] because the analysis was performed prenatally.

The phenotype of trisomy 5p usually includes psychomotor retardation and a characteristic facies, and the pregnancy is often complicated by polyhydramnios. The latter was present in the two cases published by D’amato Sizonenko et al. [9], in the case reported by Avansino et al. [7] and in the case reported by Armstrong et al. [4]. The pregnancy of our patient wasn’t complicated by polyhydramnios.

Whereas congenital heart defects have been reported in only three cases [7, 10, 11], respiratory issues and recurrent respiratory infections have been a consistent problem for approximately 50% of the patients [4, 7, 9, 11]. In fact, as in the case of our patient, both of the patients mentioned by D’Amato Sizonenko et al. 9, were born with respiratory distress and during the first years of life they were hospitalized several times due to recurrent respiratory infections. The patient 1 [9] died at the age of 3 years 3 months as a result of respiratory failures. The patient mentioted by Armstrong et al. (2018) [4] at birth, before being discharged, spent 5 weeks in the hospital because of respiratory problems. The patient of Loscalzo et al. (2008) [11] in her additional medical complications have included multiple hospital admissions related to respiratory issues including laryngomalacia.

73% of patients have developmental delay [4, 7, 9, 11–13], while 63% have intellectual disability [4, 8–10, 12, 13].

82% of patients presented macrocephaly [4, 7, 9–11, 13, 14], 91% have hypertelorism [7–14], 63% have upslanted palpebral fissures [7–9, 11, 14], 54% have midface hypoplasia [4, 7, 9, 13] and 73% have depressed nasal bridge [7–9, 11, 13, 14].

The clinical features described in our patient are concordant with many of the features presented by the patients of the ten cases collected in Table 1, especially the ID, macrocephaly, and the dysmorphic features.

**Fig. 4:**
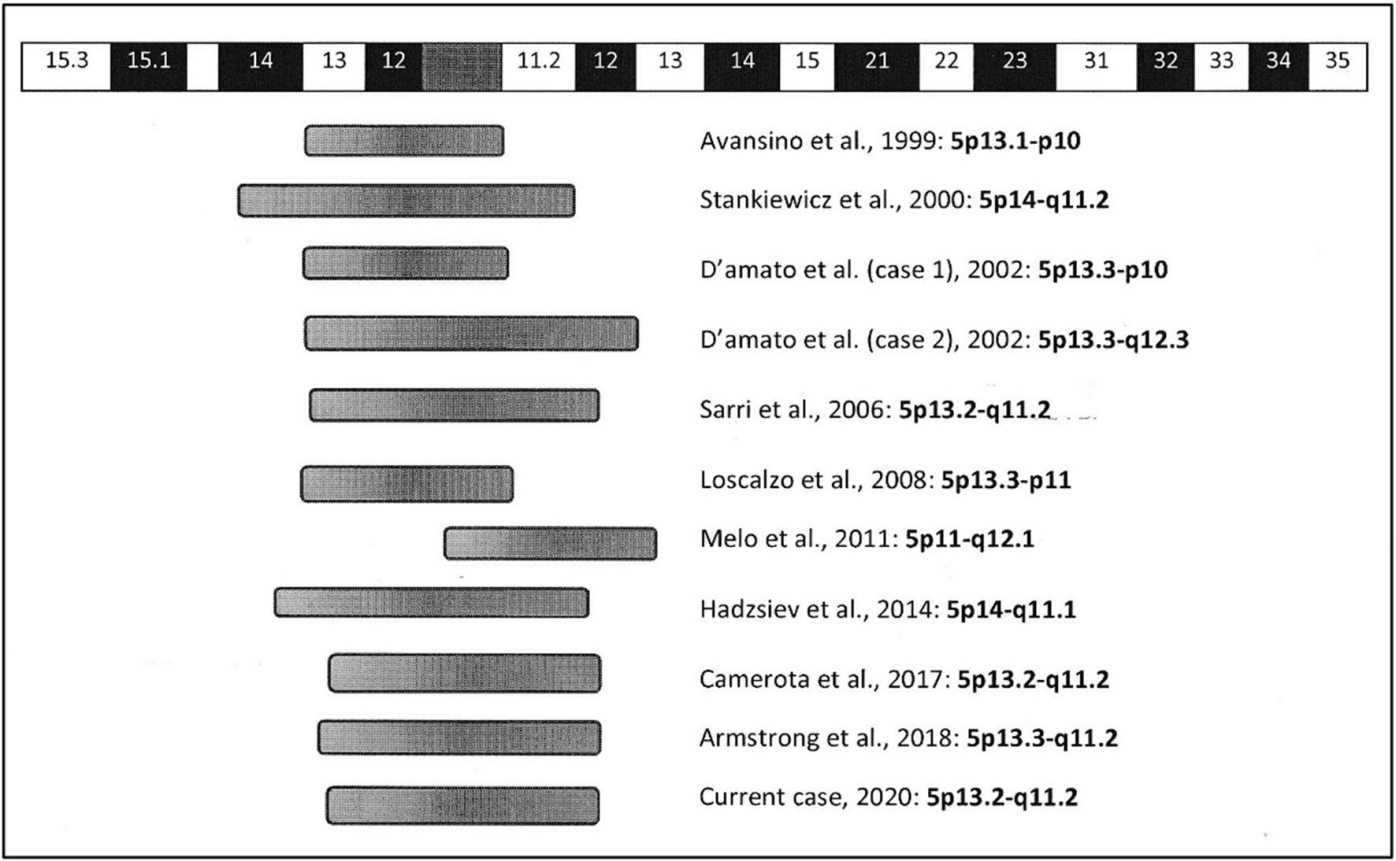
Schematic representations of sSMC5 size of 11 cases present in literature

The approximative breakpoints/genetic content of the sSMC5 documented with or without clinical findings and with an adequate cytogenetic characterization are shown in Fig. 4.

All the cases reported involved partial trisomy of 5p-arm. A critical region has been proposed for 5p13, which, in trisomy, is associated with pregnancy complicated by polyhydramnios, psychomotor delay and characteristic facial features. Of the ten reported cases with a molecular cytogenetic content overlapping our case, six involve 5p-arm euchromatic content, namely, the critical region proposed for 5p13, and euchromatic material from 5q-arm (Stankiewicz et al. [5] (case 1); D’Amato Sizonenko et al. [9] (case 2); Sarri et al. [10]; Hadzsiev et al. [13]; Camerota et al. [14] and Armstrong et al. [4]). The dysmorphic features of these cases are concordant with the ones described for trisomy involving 5p13 (Avansino et al. [7]; D’Amato Sizonenko et al. [9] (case 1)).

Therefore, even if the sSMC5’s clinical phenotype is well-defined with ID, macrocephaly and characteristic dysmorphisms, a genotype/phenotype correlation is difficult due to breakpoint distribution heterogeneity and to different levels and distributions of mosaicism [12].

For chromosome 8 we compared 18 cases present in the literature with our clinical case, as reported in Table 2a, b.

**Table 2:** Genetic and clinical features of cases with sSMC8

	Allen and Hodgkin [15]	Biennow et al. [16]	Digilio et al. pz 1 [17]	Digilio et al. pz 2 [17]	Melnyk and Dewald [18]	Ohashi et al. [19]	Butler et al. [20]	Spinner et al. [21]	Rothernmund et al. II-1 [22]	Rothernmund et al. III-1 [22]	Rothenmund et al. III-2 [22]	Present case	Total
(a)													
sSMC coordinates	8p21-pter	centromeric	Isodicentric 8p;8p	Isodicentric 8p;8p	8p11.2-q11.2	(8p23.1-pter)	pericentric	Pericentric p11-q11	pericentric	pericentric	pericentric	8p11.21q11.21	
Karyotype	46 XX -11+der (11)t(8;11) (p21q25)	46 XX /47XX+mar	46XX/46XX-8+idic(8) (qter-p23)	46XX/46XX-8+idic(8) (qter-p23)	47 XX+mar	47 XX+mar	47 XY+mar	47 XY+mar	46 XY (90) /47XY+mar (10)	47 XX+mar	47 XX+mar	48, XX,+mar1,+mar2	
Age at evaluation	At birth	1 year	14 months	2 months	15 months	2 years	At birth	7 months	30 years	4 years	XX at birth	XX, 21 months	
Gestational age	34 weeks	39 weeks	28 weeks	At term	At term	35 weeks	At term	At term	/	At term	At term	30 weeks	
Developmental delay	−	−	−	+	+	+	−	+	−	+	+	+	11/19
Intellectual disability	+	+	+	+	+	−	+	+	−	+	+	+	16/19
Congenital heart defect	+	−	+	+	−	+	−	−	−	−	+	+	7/19
Respiratory issues	−	−	+	−	−	+	−	−	−	+	−	+	6/19
Macrocephaly	+	−	−	−	−	−	−	+	−	−	−	+	5/19
Upslanted palpebral fissures	−	−	−	+	+	−	−	−	−	−	−	+	4/19
Hypertelorism	+	+	−	+	−	−	−	−	−	−	−	+	5/19
Midface hypoplasia	−	−	−	−	−	−	−	−	−	−	−	+	1/19
Depressed nasal bridge	−	−	+	+	+	−	−	−	−	−	−	+	5/19

	Batanian et al. pz 1 [23]	Batanian et al. pz 2 [23]	Batanian et al. pz 3 [23]	Tonk et al. pz 1 [24]	Tonk et al. pz 2 [24]	Loeffle et al. [25]	Vander Pluym et al. [26]	Present case	Total				
(b)													
sSMC coordinates	8cen-p12	8cen-p21	Dup (8cen-p21)	pericentric	pericentric	8p12-q12	8p11.21q11.21	8p11.21q11.21					
Karyotype	46 XY/47 XY +mar	47 XX +mar	46,XX /47,XX+mar	47 XX+mar	46,XY /47,XY+mar	46 XX/47 XX +mar	47 XX, +mar	48, XX,+mar1,+mar2					
Age at evaluation	5 years	10 years	At birth	7 months	6 months	16 years	3 years	21 months					
Gestational age	At term	At term	37 weeks	At term	At term	?	At term	30 weeks					
Developmental delay	+	+	−	−	+	−	+	+	11/19				
Intellectual disability	+	+	−	+	+	+	+	+	16/19				
Congenital heart defect	−	+	−	−	−	−	−	+	7/19				
Respiratory issues	+	−	−	−	−	−	+	+	6/19				
Macrocephaly	−	+	−	−	+	−	−	+	5/19				
Upslanted palpebral fissures	−	−	−	−	−	+	−	+	4/19				
Hypertelorism	−	−	−	−	−	+	−	+	5/19				
Midface hypoplasia	−	−	−	−	−	−	−	+	1/19				
Depressed nasal bridge	−	−	+	−	−	−	−	+	5/19				

.

Of all the cases present in the literature, we have considered only 18 and of these the 84% present intellectual disability [15–18, 20–26] and 58% have developmental delay [17–19, 21–24, 26].

Concerning the dysmorphic features, all patients have some, but these features are very widely and not in agreement with those that our patient present.

Only 26% of patients presented macrocephaly [15, 21, 23, 24] and have hypertelorism [15–17, 25], 21% have upslanted palpebral fissures [17, 18, 25]^.^ No one have hypoplasia of the midface and only 26% present depressed nasal bridge [17, 18, 23].

In 37% of the cases, we found some heart congenital defections included VSD with coarctation of aorta [15], valvular pulmonary stenosis with a secondum ASD [17, case 1], VSD with persistent left superior vena cava [17, case 2], patent ductus arteriosus with pulmonary hypertension [19], coarctation of aorta [22] and anomalous pulmonary venous return [23].

In 32% of the cases, there are respiratory problems such as respiratory distress at the birth [17], recurrent respiratory infections [19], recurrent otitis [22, 23, 26], Osas [23] and asthma [26].

In conclusion, we report the first case with two markers: one from chromosome 5 and one from chromosome 8. Based on the data reported, we can affirm that the phenotype of our patient is probably caused mainly by the presence of the sSMC.

## Data Availability

The data that support the finding of this study are available from the corresponding author.

## Abbreviations

sSMC: Small supernumerary marker chromosome
sSMC5: Small supernumerary marker chomosome 5
sSMC8: Small supernumerary marker chromosome 8
inv dup: Inverted duplicated chromosome
min: Minute chromosome
R: Ring chromosome
der: Derivative chromosome
ADHD: Attention deficit hyperactivity disorder
FISH: Fluorescent in situ hybridization analysis
EEG: Electroencephalography
MRI: Magnetic resonance imaging
IRRs: Recurrent respiratory infections
CF: Cystic fibrosis

## References

[1] Liehr T, Claussen U, Starke H (2004). Small supernumerary marker chromosomes (sSMC) in humans. Cytogenet Genome Res.

[2] Liehr T (2011). Small supernumerary marker chromosomes (sSMC), a guide for human geneticists and clinicians.

[3] Liehr T, Mrasek K, Weise A, Dufke A, Rodriguez L, Martinez Guardia N, Sanchis A, Vermeesch JR, Ramel C, Polityko A, Haas OA, Anderson J, Claussen U, von Eggeling F, Starke H (2006). Small supernumerary marker chromosomes—progress towards a genotype-phenotype correlation. Cytogenet Genome Res.

[4] Armstrong ME, Weaver DD, Lah MD, Vance GH, Landis BJ, Ware SM, Helm BM (2018). Novel phenotype of 5p13.3-q11.2 duplication resulting from supernumerary marker chromosome 5: implications for management and genetic counseling. Mol Cytogenet.

[5] Shao H-Y, Miao Z-Y, Liu X-Y, Hou X-F, Hong Wu (2011). Molecular cytogenetic characterization of mosaicism for a small supernumerary marker chromosome derived from chromosome 8 associated with congenital hypoplasia of the tongue and review of the literature. Taiwan J Obstet Gynecol.

[6] Liehr T. Small supernumerary marker chromosomes. 2021. http://cs-tl.de/DB/CA/sSMC/0-Start.html.

[7] Avansino JR, Dennis TR, Spallone P, Stock AD, Levin ML (1999). Proximal 5p trisomy resulting from a marker chromosome implicates band 5p13 in 5p trisomy syndrome. Am J Med Genet.

[8] Stankiewicz P, Bocian E, Jakubow-Durska K, Obersztyn E, Lato E, Starke H (2000). Identification of supernumerary marker chromosomes derived from chromosomes 5, 6, 19 and 20 using FISH. J Med Genet.

[9] D’amato Sizonenko LD, Ng D, Oei P, Winship I (2002). Supernumerary marker chromosome 5: confirmation of a critical region and resultant phenotype. AM J Med Genet.

[10] Sarri C, Gyftodimou Y, Grigoriadou M, Pandelia E, Kalogirou S, Kokotas H, Mrasek K, Weise A, Petersen MB (2006). Supernumerary marker chromosome 5 diagnosed by M-FISH in a child with congenital heart defect and unusual face. Cytogenet Genome Res.

[11] Loscalzo ML, Becker TA, Stucliffe M (2008). A patient with an interstitial duplication of chromosome 5p11-p13.3 further confirming a critical region for 5p duplication syndrome. Eur J Med Genet.

[12] Melo JB, Backx L, Vermeesch JR, Santos HG, Sousa AC, Kosyakova N (2011). Chromosome 5 derived small supernumerary marker: toward a genotype/phenotype correlation of proximal chromosome 5 imbalances. J Appl Genet.

[13] Hadzsiev K, David D, Szabò G, Czakò M, Melegh B, Kosztoalanyi G (2014). Partial trisomy of the pericentromeric region of chromosome 5 in a girl with binder phenotype. Cytogenet Genome Res.

[14] Camerota L, Pitzianti M, Postorivo D, Nardone A, Ligas C, Moretti C (2017). A small supernumerary marker derived from the pericentromeric region of chromosome 5: case report and delineation of partial trisomy 5p phenotype. Cytogenet Genome Res.

[15] Allen EF, Hodgkin WE (1983). Trisomy for 8p21 leads to pter owing to a familiar translocation. J Med Genet.

[16] Blennow E, Annerén G, Bui TH, Berggren E, Asadi E, Nordenskjöld M (1993). Characterization of supernumerary ring marker chromosomes by fluorescence in situ hybridization (FISH). Am J Hum Genet.

[17] Digilio MC, Giannotti A, Florida G, Uccellatore F, Mingarelli R, Danesino C (1994). Trisomy 8 syndrome owing to isodicentric 8p chromosomes: regional assignment of a presumptive gene involved in corpus callosum development. J Med Genetic.

[18] Melnyk AR, Dewald G (1994). Identification of a small supernumerary ring chromosome 8 by fluorescent in situ hybridization in a child with developmental delay and minor anomalies. Am J Med Genet.

[19] Ohashi H, Wakui K, Ogawa K, Okano T, Niikawa N, Fukushima Y (1994). A stable acentric marker chromosome: possible existence of an intercalary ancient centromere at distal 8p. Am J Hum Genet.

[20] Butler MG, Roback EW, Ga A, Dev VG (1995). Identification of a ring chromosomes as a ring 8 using fluorescent in situ hybridization (FISH) in a child with multiple congenital anomalies. Am J Med Genet.

[21] Spinner NB, Grae KR, Owens L, Sovinsky L, Pellegrino JE, McDonald-McGinn D (1995). Mosaicism of chromosome 8-derived minute marker chromosome in a patient with manifestations of trisomy 8 mosaicism. Am J Med Genet.

[22] Rothenmund H, Chudley AE, Dawson AJ (1997). Familiar transmission of a small supernumerary marker chromosome 8 identified by FISH: an update. AM J Med Genet.

[23] Batanian JR, Huang Y, Gottesman GS, Grange DK, Blasingame AV (2000). Preferential involvement of the short arm in chromosome 8-derived supernumerary markers and ring as identified by chromosome arm painting. Am J Med Genet.

[24] Tonk VS, Kukolich MK, Morgan D, Khan A, Jalal SM (2000). Ring chromosome 8 syndrome: further characterization. Am J Med Genet.

[25] Loeffler J, Soelder E, Erdel M, Utermann B, Janecke A, Duba H-C, Utermann G (2003). Muellerian aplasia associated with ring chromosome 8p12q12 mosaicism. Am J Med Genet.

[26] Juliana H, Pluyl V, O’Sullivan J, Andrew G, Bolduc FV (2003). Genomic characterization of a chromosome 8 pericentric trisomy. Clin Case Rep.

